# Direct and indirect effects of banker plants on population establishment of *Harmonia axyridis* and aphid control on pepper crop

**DOI:** 10.3389/fpls.2022.1083848

**Published:** 2022-12-12

**Authors:** Jie Wang, Yajie Yang, Yuanxi Li, Zhenyu Jin, Nicolas Desneux, Peng Han, Su Wang, Shu Li

**Affiliations:** ^1^ Institute of Plant Protection, Beijing Academy of Agriculture and Forestry Sciences, Beijing, China; ^2^ Department of Entomology, College of Plant Protection, Nanjing Agricultural University, Nanjing, China; ^3^ Forewarning and Management of Agricultural and Forestry Pest, Hubei Engineering Technology Center and College of Agriculture, Yangtze University, Jingzhou, China; ^4^ Université Côte d’Azur, INRAE, CNRS, UMR ISA, Nice, France; ^5^ Yunnan Key Laboratory of Plant Reproductive Adaptation and Evolutionary Ecology /Institute of Biodiversity, School of Ecology and Environmental Sciences, Yunnan University, Kunming, China

**Keywords:** aphid, banker plants, IPM, indirect interaction, population quantitative relationship

## Abstract

Banker plant systems increase biological pest control by supporting populations of natural enemies, i.e., using non-pest arthropod species as alternative prey. However, the presence of alternative prey does not always result in improved control of the target pest species owing to the complexity of biotic interactions. To increase the effectiveness of banker plants in IPM programs, a fine understanding of the indirect interactions between target aphid and alternative prey mediated by biocontrol agents is necessary. In this study, we first established a banker plant system, banker plant (*Vicia faba*)*–*alternative prey (*Megoura japonica*)*–*predator (*Harmonia axyridis*), to control the target pest (*Myzus persicae*) on pepper. We found that *M. japonica* strongly preferred faba bean as a host plant and posed no risk to *Solanaceous* crops. *Harmonia axyridis* adults had no significant predation preference for the alternative prey. In the short term, the interaction direction of the two aphid species depended on the relative initial density and the timescale. *Harmonia axyridis* showed a stronger negative effect on *M. persicae* than that on *M. japonica*. In the long term, the presence of alternative prey, *M. japonica*, enhanced the control effect of *H. axyridis* to *M. persicae* with initial density of 100–500 aphids per plant. The presence of the alternative prey could proliferate the population of *H. axyridis*, with from 0.2- to 2.1-fold increase of *H. axyridis* eggs. Overall, we put forward a strategy for setting the initial density of alternative prey of the banker plant system to target the high and low density of aphids, which highlighted the importance of indirect interactions in designing a proper banker plant system.

## Introduction

Conservation biological control (CBC) could enhance the survival, longevity, and fecundity of natural enemies by habitat management to increase their effectiveness in pest control (Gurr et al., 2017). The key to an effective CBC is an early colonization and establishment of natural enemies in crops, when pest populations are still at low densities (Symondson et al., 2002). Different types of functional plants have been proposed to support natural enemies, increasing the efficiency and sustainability of biological control of pests (Xie et al., 2012; Zhao et al., 2017; Hatt et al., 2018; Hatt et al., 2019b; Damien et al., 2020). As an important form of CBC, banker plant system could preserve populations of beneficial arthropods in crops by providing alternative prey/hosts and could provide an on-farm refuge for spontaneous populations for sustainably effective pest control (Frank, 2010; Huang et al., 2011; Li et al., 2015). The use of banker plant systems has been increasingly investigated and developed for greenhouse and field crops (Zheng et al., 2017; Xu et al., 2020; Chen et al., 2022). However, research studies mainly focus on the establishment of the banker plant system (Wang et al., 2020; Wang et al., 2021), and further research is needed to understand the quantitative relationship among trophies when the banker plant system applied to pest control (Li et al., 2020).

The population dynamic of species in ecosystem not only depends on the direct feeding behavior in food web (Symondson et al., 2002) but also on the indirect interaction among species (Montoya et al., 2009; Zhang et al., 2019). In agroecosystem, preys without direct resource competition could have indirect interaction mediated by shared natural enemies (van Veen et al., 2006; Frost et al., 2016; Holt and Bonsall, 2017). These interactions affect the predator’s predation in the short term (Desneux and O’Neil, 2008; Jaworski et al., 2013) and even direct the predator and prey population dynamics in the long term (e.g., apparent competition, Holt, 1977; Jaworski et al., 2015; Desneux et al., 2019). Understanding how pests, alternative prey, and natural enemy interact in complex managed environments is essential to the pest management in agriculture (Chailleux et al., 2014). When the banker plant system is used to control the target pests in the field, there is no direct resource competition between the alternative prey and the target pests. Whether the indirect interaction mediated by the shared natural enemies affects the population dynamics is still uncovered.

Depending on the temporal or spatial scale, the behavior of prey, and the quality and density of prey, the natural enemy–mediated indirect interactions can take different forms, such as apparent competition, mutualism, amensalism, and commensalism (Chaneton and Bonsall, 2000; Chailleux et al., 2014). Natural enemy–mediated indirect interactions contribute to pest population dynamics, and insufficient efforts have been made to generate predictions that would facilitate the use of indirect interactions in biological control in different spatial scales (Chailleux et al., 2014). On a large scale, maintaining a high level of biodiversity has been proved to promote the growth of natural enemies of pests and improve the control of target pests (Karp et al., 2018). Small-scale experiments show that natural enemies benefit from mixed food by alternative prey (Liu et al., 2006; Messelink et al., 2008). Many studies have also shown that alternative prey/food can help natural enemy populations establish in crops before pest arrival (Gurr et al., 2016; Jaworski et al., 2019; Sanchez et al., 2021). Whereas alternative prey could induce a decrease in pest densities in crops through indirect interactions with a shared natural enemy (i.e., apparent competition) (Liu et al., 2006; Desneux and O’Neil, 2008), generalist predators are widely used as biocontrol agents to regulate populations of pest in agriculture (Symondson et al., 2002; van Lenteren, 2012). When the alternative prey is applied to pest biological control programs, it is necessary to clarify the indirect interactions induced by the generalist predators (Chailleux et al., 2014; Costa and Anjos, 2020).

This study aims to identify the quantitative relationship among trophies when the banker plant system applied to pest control. Targeting the widely distributed and important pest of protected vegetables, *Myzus persicae* (Aparicio et al., 2020; Wang et al., 2022), we explored the interactions when the *Harmonia axyridis* banker plant system was applied to *M. persicae* control. We hypothesized that (1) *Harmonia axyridis* adults do not show predation preference for the two aphid species; (2) the direction, sign, and strength of the indirect interactions mediated by shared predator between the two aphid species depends on the relative initial density and time; (3) the alternative prey, *M. japonica*, preserve populations of *H. axyridis* and provide sustainably effective pest control in caged study.

## Materials and methods

### Insects and plants

Initial colonies of the green peach aphid (*Myzus persicae*), the bean aphid (*Megoura japonica*), and the harlequin ladybird (*Harmonia axyridis*) were collected from Beijing Noah Organic Farm (116°59′E, 40°6′N), Beijing, China. The aphids, *M. persicae*, was reared on *Capsicum annuum* var. Zhongjiao 105 (Institute of Vegetables and Flowers, CAAS), and *M. japonica* was reared on *Vicia faba* var. Kexi (Sichuan Kexi Seed Co., Ltd.). The ladybeetle *H. axyridis* was reared in cages (35 × 35 × 55 cm) made of nylon mesh net and aluminum alloy frames. They were fed *ad libitum* with *M. japonica* reared on faba bean plants. All colonies were kept at 25 ± 2°C, 60 ± 5% relative humidity (RH), 16-hour light: 8-h dark (L16:D8-h) photoperiod in the insectary of the Institute of Plant Protection, Beijing Academy of Agriculture and Forestry Sciences (BAAFS). The culture environment was regulated using an automatic environmental management system (LT-100, Suntech, Beijing, China).

The tomato, eggplant, and pepper seedlings were grown in plastic trays (54 × 28 × 6 cm, 21 plants per tray) and transplanted individually in plastic pots once they reached 15 cm in height. All plants were cultured individually in plastic pots (H = 25 cm, D = 15 cm) with commercial substrate (Pindstrup^®^) at 25 ± 2°C, 60 ± 5% RH, and L16:D8-h photoperiod. The tomato, eggplant, and pepper plants reaching 30–35 cm in height with five to seven fully expanded true leaves, and faba bean plants with five to seven fully expanded true leaves were used for experiments.

### Evaluating *Megoura japonica* fitness on faba bean

Newborn nymph (<12 h) of *Megoura japonica* were placed individually on the back of faba bean leaves in plastic Petri dish (H = 1.5 cm, D = 5 cm). The leaves bases were covered with absorbent cotton to moisturize. Dishes were kept in the laboratory at 25 ± 2°C, 60 ± 5% RH, and L16:D8 h. The leaves were replaced every 2 days. Development and survival data were recorded daily. The presence of an exuvium was used as the criterion for molting to the next developmental stage. After the emergence of adults, the number of newborn nymphs was recorded and the nymphs were removed from the dishes daily until the death of the adult. One hundred newborn nymphs were used for the life table study (*n* = 100).

### Host specificity of *Megoura japonica*

A choice experiment was conducted to evaluate the host specificity of *M. japonica* to the faba bean and three *Solanaceous* vegetables (tomato, eggplant, and pepper). One 2.5-cm (in diameter) leaf disc of each plant was placed on the wet filter paper with an equal distance between them to form a square in a plastic Petri dish (H = 1.5 cm, D = 9 cm). Thirty third-instar nymphs of *M. japonica* were released onto the center of the filter paper. Dishes were kept in the laboratory at 25 ± 2°C, 60 ± 5% RH, and L16:D8 h. The number of *M. japonica* on each leaf disc (treatment) was counted at 3, 6, 12, and 24 h after the release of aphids into the Petri dish. The position of different leaf discs was determined randomly and rotated in each replication. The experiment was a randomized complete block design and replicated 16 times (*n* = 16).

A survival experiment was conducted to determine survival or development of *M. japonica* on different host plants (faba bean, tomato, eggplant, and pepper). Ten fourth-instar nymphs of *M. japonica* were introduced to the head of the potted plant with five to seven fully expanded true leaves. Each plant was placed randomly at an interval of 3 m in a greenhouse. The greenhouse was maintained at 26 ± 2°C, 70 ± 10% RH, and L16:D8 h. The number of *M. japonica* that survived on each plant of different host plants was recorded at 24, 48, 72, and 96 h. Each treatment (host plant) was replicated 10 times (*n* = 10).

### Predation preference of *Harmonia axyridis* to *Myzus persicae* and *Megoura japonica*

On the basis of the daily predation of *H. axyridis* adults on *M. persicae* and *M. japonica*, we set five levels of prey complex. The total number of preys per dish remained constant at 180. The ratios tested of *M. persicae* and *M. japonica* were 180:0, 120:60, 90:90, 60:120, and 0:180. In each experimental unit, third-instar nymphs of *M. persicae* and *M. japonica* were randomly chosen from the stock colony reared in the laboratory and carefully transferred onto a Petri dish (9 cm in diameter) using a fine brush for 1 h of acclimatization, after which one newly hatched (< 24 h) adult of *H. axyridis* was added to the center of the dish. Male and female *H. axyridis* were tested separately. All adults suffered starving for 24 h before being used in preference experiments. The experiments were conducted in the laboratory (25 ± 1°C, 65 ± 5% RH, and L16:D8 h). After 24 h, each prey type consumed by the predator was counted. Ten replicates were prepared for each prey complex (*n* = 10).

### Interaction between two aphids when the application of banker plant system

**Figure 1:**
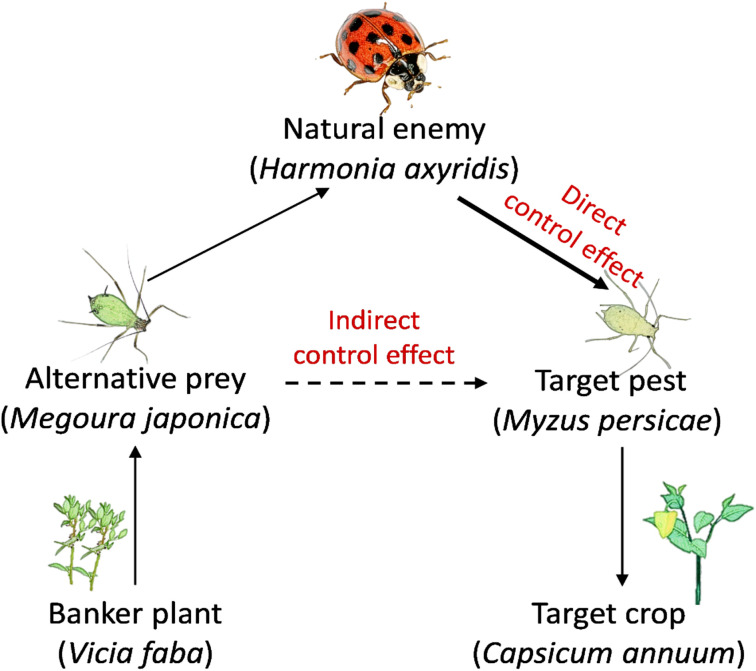
The scheme of the interaction of the predator (*Harmonia axyridis*), alternative prey (*Megoura japonica*), and target pest (*Myzus persicae*) when the banker plant system applied to biological aphid control on pepper.

In greenhouse, we studied the dynamic relationship, interaction direction, sign, and strength of the predator (*H. axyridis*), alternative prey (*M. japonica*), and target pest (*M. persicae*) when banker plant system applied to *M. persicae* control using a 2 x 2 factorial design (**Figure 1**). The first three-level treatment varied the alternative prey density (0, 200, and 400 per plant). The second three-level treatment varied the target pest density (100, 300, and 500 per plant) in the microcosms. One potted pepper and faba bean were set oppositely in a microcosm (35 × 35 × 55 cm). Third-instar nymphs of *M. persicae* and *M. japonica* were randomly chosen from the colonies reared in the laboratory and released on the pepper and faba bean plant, respectively. After 1 day of acclimatization, one pair newly hatched (<24 h) adults of *H. axyridis* was released. Population dynamics of the three insect species were monitored 39 days following the introduction of predators. Adults and nymphs of *H. axyridis*, *M. persicae*, and *M. japonica* were counted on all leaves every 3 days. Plants were fertilized and watered to avoid any abiotic stress. Each treatment was repeated 15 times (*n* = 15).

### Data analysis

According to Özgökçe et al. (2018), the life table raw data were analyzed using the computer program TWOSEX-MSChart (Chi, 2017b) based on the age-stage, two-sex life table theory (Chi and Liu, 1985; Chi, 1988). The variances and standard errors of the developmental time, fecundity, longevity, and population parameters were estimated using the bootstrap technique (Efron and Tibshirani, 1993) with 100,000 resampling. The bootstrap routine is embedded in the TWOSEX program. The difference between treatments was examined by using paired bootstrap test (Efron and Tibshirani, 1993).

The data obtained from the host specificity experiments did not follow normal distribution (Shapiro–Wilk test, *P<* 0.05) and/or homoscedasticity (Bartlett’s test, *P<* 0.05), and Friedman test and Kruskal–Wallis test was used to compare the differences among treatments for the choice rate of *M. japonica* on different plants in laboratory choice experiments and number of *M. japonica* colonizing on different plants in greenhouse, respectively.

Manly’s model was used to estimate the predation preference of *H. axyridis* to *M. japonica* and *M. persicae* at different prey ratios. Manly (1973) equation:


$$\beta j=\frac{\text{ln}(r_{\text{j}}/A_{j})}{\sum_{i=1}^{n}\text{ln}(r_{i}/A_{i})}$$

*A_i_* was the number of individuals of a given prey type *i* available for predation by *H. axyridis* (= total number of prey available for predation) and *r_i_* was the number of a prey type *i* that has not been attacked. The number of prey types was *n* = 2 and *β_j_* = 1/n when prey was chosen randomly.

The Wilcoxon signed-rank test of two related samples in non-parametric test was used to compare the predation preference of *H. axyridis*, and the Mann–Whitney U-test of two independent samples was used to compare the difference in predation preference between male and female adults (*P<* 0.05).

To differentiate long-term from short-term interactions between preys, the data were divided into two time periods. Short-term interactions were assessed on days 0–9 and long-term interactions on days until the end of experiments according to the predation pressure in the system.

Log response ratios (RRs) were used separately for each aphid species as a measure of the strength of the negative impact of predation, and mean ratios of the log abundance of the two aphid species were used as a measure of the symmetry of the impact of predation on each aphid species (Gelman and Hill, 2007). The impacts of predation at each treatment level were compared with the control (no alternative prey, *M. japonica*). Log RRs was also used to estimate the log-proportional difference between the mean of a particular treatment level and that of a control (Hedges et al., 1999). For each experiment, the mean ratios of log abundance at each treatment level of different sampling days were tested for significant differences from the control without alternative prey using *t*-test.

A delta correction (RRΔ) and its variance [var (RRΔ)] were used on the basis of the standard deviation (SD), sample size (N), and mean (X) of the treatment (T) and control (C) following Lajeunesse (2015):


$${\text{RR}}^{\Delta }=\text{ln}\frac{\bar{X_{\text{T}}}}{\bar{X_{\text{C}}}}+\;\frac{1}{2}\left[\frac{{\left({\text{SD}}_{\text{T}}\right)}^{2}}{N_{\text{T}}{\bar{X}}_{\text{T}}^{2}}-\frac{{\left({\text{SD}}_{\text{C}}\right)}^{2}}{N_{\text{C}}{\bar{X}}_{\text{C}}^{2}}\right]$$

$$\text{var}(\text{RRΔ})=\left[\frac{{\left({\text{SD}}_{\text{T}}\right)}^{2}}{N_{\text{T}}{\bar{X}}_{\text{T}}^{2}}+\frac{{\left({\text{SD}}_{\text{C}}\right)}^{2}}{N_{\text{C}}{\bar{X}}_{\text{C}}^{2}}\right]+\frac{1}{2}\left[\frac{{\left({\text{SD}}_{\text{T}}\right)}^{4}}{N_{T}^{2}{\bar{X}}_{\text{T}}^{4}}+\frac{{\left({\text{SD}}_{\text{C}}\right)}^{4}}{N_{C}^{2}{\bar{X}}_{\text{C}}^{4}}\right]$$

As the ladybird abundance data did not follow normal distribution (Shapiro–Wilk test, *P<* 0.05) and/or homoscedasticity (Bartlett’s test, *P<* 0.05), Kruskal–Wallis test was used to compare the differences among treatments. All statistical analyses were performed using SPSS 25.0 (SPSS Inc., Chicago, IL, USA).

## Results

### *Megoura japonica* fitness on faba bean

The developmental times, longevity, reproductive periods, and fecundity of *M. japonica* on faba bean are shown in **Table 1**. The developmental time periods of the pre-adult and adult stage were 5.63 ± 0.08 days and 15.03 ± 0.95 days, respectively. The longevity and reproductive periods were 19.02 ± 0.99 days and 9.14 ± 0.53 days, respectively. The fecundity was 44.09 ± 3.34 offspring (**Table 1**).

**Table 1:** The developmental, longevity, and fecundity of *Megoura japonica* fed on faba bean.

Parameters	Development stage	n	Mean ± SE
Developmental time (days)	First instar	100	1.00 ± 0.02
	Second instar	100	1.12 ± 0.02
	Third instar	95	1.41 ± 0.03
	Fourth instar	90	2.12 ± 0.03
	Pre-adult	90	5.63 ± 0.08
	Adult	90	15.03 ± 0.95
Longevity (days)	Adult	90	19.02 ± 0.99
Reproductive period (days)	Adult	90	9.14 ± 0.53
Fecundity (offspring)	Adult	90	44.09 ± 3.34

### Host specificity of *Megoura japonica*

**Figure 2:**
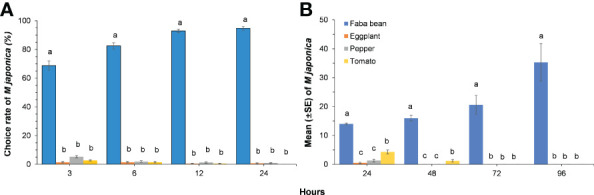
The choice rate of *Megoura japonica* on different plants over time in laboratory choice experiments **(A)** and mean (± SE) of *Megoura japonica* colonizing on different plants in greenhouse **(B)**. Means capped with different letters in the same sampling time are significantly different (*P* < 0.05; Friedman test for choice rate and Kruskal–Wallis test for mean of *M. japonica*) among treatments.

*Megoura japonica* significantly preferred faba bean leaf disc in the laboratory choice experiments (3H: *X^2^* = 39.146, *P<* 0.001; 6H: *X^2^* = 33.064, *P<* 0.001; 12H: *X^2^* = 39.992, *P<* 0.001; 24H: *X^2^* = 30.157, *P<* 0.001). The number of *M. japonica* chose faba bean increased over time, whereas the number of *M. japonica* on eggplant, pepper, and tomato decreased rapidly over time (**Figure 2A**). *Megoura japonica* developed better on the faba bean plants than that on the *Solanaceae* crops. The population size of the bean aphid on faba bean plants reached the 3.5 times of the initial value after 96 h, whereas the population size on eggplant, pepper, and tomato decreased significantly (24H: *X^2^* = 29.431, *P<* 0.001; 48H: *X^2^* = 33.056, *P<* 0.001; 72H: *X^2^* = 37.981, *P<* 0.001; 96H: *X^2^* = 37.956, *P<* 0.001). Although the number of *M. japonica* on tomato plants was significantly higher than that on eggplant and pepper at 24 h, they almost went extinction on eggplant, pepper, and tomato at 72 h, indicating that those plants were unsuitable hosts for *M. japonica* (**Figure 2B**).

### Predation preference

**Figure 3:**
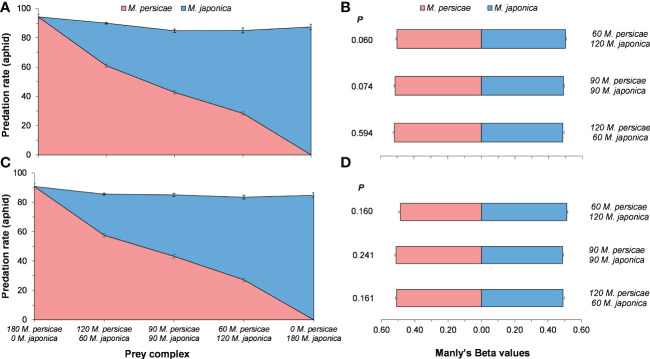
Mean (± SE) of consumed *Myzus persicae* and *Megoura japonica*, predation preference of female **(A, B)** and male **(C, D)** *Harmonia axyridis* in various prey complex. Wilcoxon signed-rank test of two related samples in non-parametric test was used to compare the predation preference of *H. axyrdis*.

Predation preference of *H. axyridis* strongly depended on prey relative abundance with a disproportionately high predation on the most abundant prey (**Figures 3A, C**). On the basis of the analyses of Manly’s Beta values (*β_j_*), predation preference did not change across various *M. persicae* and *M. japonica* relative prey ratios (**Figures 3B, D**). Female *H. axyridis* consumed a slightly higher number of prey than male, but Manly’s Beta values were no significant difference between genders (60:120 *M. persicae*:*M. japonica*, *P =* 0.560; 90:90 *M. persicae*:*M. japonica*, *P =* 0.845; 120:60 *M. persicae*:*M. japonica*, *P =* 0.46).

### Interactions between aphids in short term

**Figure 4:**
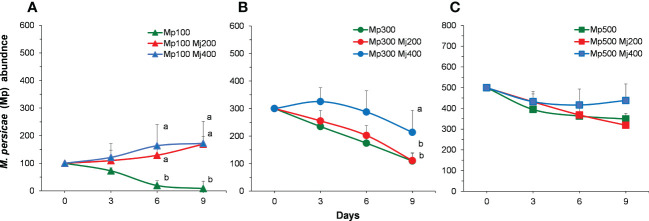
Population abundance of *Myzus persicae* (Mp) in different treatments with the initial density of *M. persicae* is 100 **(A)**, 300 **(B)**, and 500 **(C)**. Mj, *Megoura japonica*. Different letters next to the curves indicate significant differences among treatments (P<0.05).

When the initial density of *M. persicae* is low (100 aphids per plant) (i.e., the initial density of *M. persicae* is less than that of *M. japonica*.), the density of *M. persicae* is significantly different at the same time (*X* ^2^ = 22.182, *P<* 0.001) but no interaction between time (*X* ^2^ = 2.431, *P* = 0.051). The density of *M. persicae* was significantly higher than that of the control on the sixth and ninth days (6 days: *X* ^2^ = 25.068, *P<* 0.001; 9 days: *X* ^2^ = 24.227, *P<* 0.001; **Figure 4A**). When the initial density of *M. persicae* is medium (300 aphids per plant), the density of *M. persicae* among treatments is significantly different at the same time (*X* ^2^ = 6.357, *P =* 0.002). In addition, the density of *M. persicae* was significantly higher than that of the control on the ninth day (3 days: *X* ^2^ = 2.638, *P* = 0.267; 6 days: *X* ^2^ = 5.439, *P* = 0.066; 9 days: *X* ^2^ = 6.145, *P* = 0.046; **Figure 4B**). With higher initial density of *M. persicae* (500 aphids per plant), no significant difference was found among treatments (*X* ^2^ = 0.392, *P =* 0.676) and sampling times (3 days: *X* ^2^ = 0.383, *P* = 0.826; 6 days: *X* ^2^ = 0.127, *P* = 0.939; 9 days: *X* ^2^ = 1.127, *P* = 0.569; **Figure 4C**).

#### Predator-mediated interaction intensity on *Myzus persicae*

**Figure 5:**
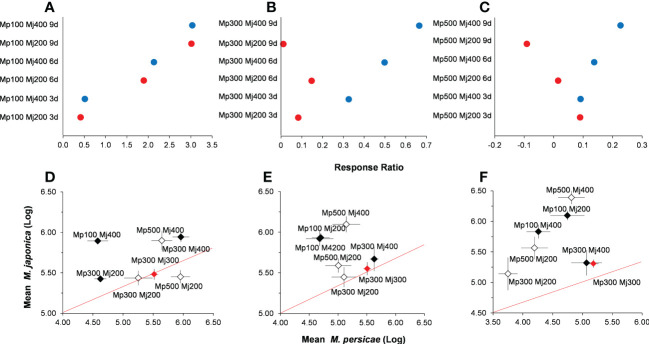
Relationships among the relative abundance of *Myzus persicae* (Mp) and *Megoura japonica* (Mj) in different treatments for log response ratios with the initial density of *M. persicae* is 100 **(A)**, 300 **(B)**, and 500 **(C)**, and sampling after 3 **(D)**, 6 **(E)**, and 9 **(F)** days.

On the basis of the density of *M. persicae* in control without alternative prey, the effect of *H. axyridis* on *M. persicae* varied from positive to negative with the increase of the initial density of *M. persicae*, and the positive effect increased as the initial density of the alternative prey, *M. japonica* (**Figure 5**). The negative impact of predation for *M. persicae* weakened with time at low initial density (**Figure 5A**). At intermediate and higher initial density, the negative impact of predation on the target prey *M. persicae* weakened with time and initial density of alternative prey (**Figures 5B, C**).

The log abundance of *M. persicae* and *M. japonica* was significantly different among treatments (**Figures 5D–F**). On day 3, the mean ratio of treatments with higher initial density of *M. persicae* (500:200 *M. persicae*:*M. japonica* and 500:400 *M. persicae*:*M. japonica*) were marginally greater than 1 (**Figures 5D–F**). Whereas the treatments with higher initial density of *M. persicae* on day 3 were significantly greater than that of the control (100:200 *M. persicae*:*M. japonica*, *P<* 0.000; 100:400 *M. persicae*:*M. japonica*, *P<* 0.001; **Figure 5D**). With the increase of time, the mean ratio of the log abundance of *M. persicae* and *M. japonica* was lower than 1 (**Figures 5D–F**). This indicated that different initial density of alternative prey had a more significant negative impact on *M. persicae* than that on *M. japonica*. Moreover, the treatments with lower initial density of *M. persicae* on days 6 and 9 were significantly greater than that of the control (**Figures 5D–F**). The mean ratio of treatment (300:200 *M. persicae*:*M. japonica*, 0.73 ± 0.01) on day 9 was significantly lower than that of the control (0.98 ± 0.02, *t* = −2.45, *P* = 0.01).

#### Effect of banker plant on *Myzus persicae* population dynamics

**Figure 6:**
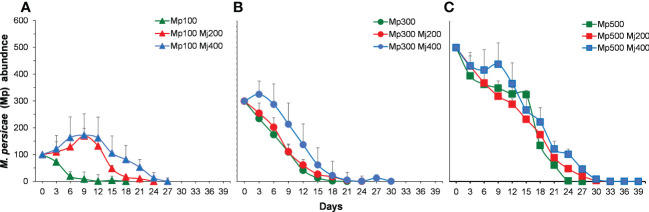
Population abundance of *Myzus persicae* (Mp) in different treatments through the whole term with the initial density of *M. persicae* is 100 **(A)**, 300 **(B)**, and 500 **(C)**. Mj, *Megoura japonica*.

Overall, a higher number of *M. persicae* were registered throughout the duration of the experiment with than that without alternative prey (**Figure 6**). At low initial density, the population of *M. persicae* in control decreased and subsided on the 18th day. Whereas the population of *M. persicae* in treatments with alternative prey increased and then decreased, reaching the peak on the ninth day, which was 1.7 times of the initial density (**Figure 6A**). The population of *M. persicae* in control at intermediate and higher initial density decreased and subsided before the treatments with *M. japonica* (**Figures 6B, C**).

#### Effect of the banker plant system on *Harmonia axyridis* abundance

*Harmonia axyridis* was sustained by the alternative prey and varied with different initial density in the system (**Figure S1**). The predator egg abundance was positively related to the total initial density of aphids (*F*= 42.893, *df* =11,133, *P<* 0.001; **Figure S2A**). In addition, the total initial density of aphids positively affected the hatching rate and net reproductive rate of *H. axyridis* egg (*F* = 7.673, *df* =11,133, *P*=0.006; **Figure S2B**; *F*= 31.824, *df* =11,133, *P<* 0.001; **Figure S2C**).

## Discussion

The banker plant system, integrating the essence of augmentative and CBC, has been widely used to aid establishment, development, and dispersal of beneficials employed in pest biological control (Parolin et al., 2012; Miller et al., 2018; Wang et al., 2021; Ardanuy et al., 2022). However, the size of the founder population of the banker plant system has received little attention despite their importance in biological control efficacy and adoption (Sanchez et al., 2021). In this study, we explored the dynamic relationship and interaction degree of the predator (*H. axyridis*), alternative prey (*M. japonica*), and target pest (*M. persicae*). We found that the interaction direction of the two aphids depends on the relative initial density and time. *Harmonia axyridis* showed a stronger negative effect on *M. persicae* than that on *M. japonica*. *Megoura japonica* with different initial densities had different effects on the proliferation of *H. axyridis*. During the whole experimental period, the presence of alternative prey, *M. japonica*, enhanced the control effect of *H. axyridis* to *M. persicae* with initial density of 100–500 aphids per plant.

Alternative prey generally has been shown to enhance predator densities and to have a negative effect on the population of another prey species, resulting in increased biocontrol services on target pests in agroecosystems (Desneux and O’Neil, 2008; Eubanks and Finke, 2014; Ramirez and Eubanks, 2016; Wang et al., 2021). When alternative prey and target pests co-existed, the spatial location of the two prey species may reduce the efficiency of indirect interactions, especially likely when the alternative prey is provided by using mulch or banker plants (Chailleux et al., 2014). Therefore, understanding how alternative prey and beneficials interact when banker plant was used is essential for pest management programs. On the timescale of this study, the interaction direction of the two aphids is related to the relative initial density and time. *Harmonia axyridis* showed a stronger negative effect on *M. persicae* than that on *M. japonica*. However, the highest hatching rate of *H. axyridis* eggs is 26.12%, which indicates that intraguild predation (IGP) may occur in the system. Previous experience of cannibalism did not increase further cannibalism frequency of *H. axyridis* (Ovchinnikov et al., 2019). Some studies have shown that intraspecific predation would negatively affect the predator numerical response, resulting in different forms indirect interactions (Holt and Lawton, 1994; Liu et al., 2006). Conversely, supplementing non-prey food resources, such as floral resources (e.g., marigold), could reduce IGP of *H. axyridis* in agroecosystems (Liang et al., 2022).

Predators could affect the colonization and abundance of a single prey by direct predation (i.e., consumptive effects) and by altering prey activity, behavior, and development (i.e., non-consumptive effects) (Orrock et al., 2008). Moreover, predator’s habit (e.g., predation preference) could also modify the strength, the direction, and the symmetry of indirect interactions among preys (Holt and Bonsall, 2017). If the predator has no preference, then the indirect interaction mediated by shared predator among preys would change from negative to positive due to the predator satisfaction or prey switching (Abrams and Matsuda, 1996; Jaworski et al., 2015). As predator preference leads to apparent competition, a critical first step was to establish if there was predator preference between the two prey species (Jaworski et al., 2013; Emery and Mills, 2020). Our results indicated that *H. axyridis* adults show no preference for the two aphid species when their densities varied in a short period (i.e., 24 h).

The RRs could quantify the effect size of predation prey individually in proportion to their abundance in the controls. This also served as an initial indicator of potential asymmetrical effects between the two aphid species (Desneux et al., 2019; Emery and Mills, 2020). We found that the interaction direction and intensity of *H. axyridis* predation on the two aphids in the system were related to the relative initial density of the two aphid species and timescales. The negative effect of *H. axyridis* on *M. persicae* was stronger than that of *M. japonica* in the short term (9 days). In addition, consistent with the results of the study by Abrams and Matsuda (1996), we found that the interaction direction between the two aphid species changed with different initial density ratio. The *t*-test of the mean ratios of the log abundance of two preys could be used to test for asymmetry in comparison with the mean ratios of the control treatments (Emery and Mills, 2020). Nevertheless, we used the *t*-test with control without alternative prey and found that the effect of *H. axyridis* on *M. persicae* changed from positive to negative with the increase of the initial density of *M. persicae*, and the positive effect increased with the increase of the initial density of *M. japonica*.

The asymmetric interaction between the two aphids mediated by *H. axyridis* may be related to the predation rate on different aphids or the asymmetric non-consumption effect of prey (Emery and Mills, 2020). We found that, although the initial density of *M. persicae* is high, the alternative prey generally trended to upward and the *M. persicae* shows a downward trend in the long term. Although *H. axyridis* adults did not show predation preference in Petri dishes, we do not take in consideration the effect of living plants on predation preference. Studies have shown that plant morphology impacts the activity and prey availability of predatory ladybugs (Grevstad and Klepetka, 1992). According to the experimental observation, *Myzus persicae* is the apical meristem scattered in pepper plants, whereas *M. japonica* are evenly distributed in faba bean plants, which may lead to differences in predation on different aphids, causing the asymmetric effect of *H. axyridis* impact on two aphids. Although the literature on short-term interaction has received less attention, it may have negative effects on biological control (Bompard et al., 2013; Chailleux et al., 2014; Blubaugh et al., 2018; Emery and Mills, 2020). Our study confirmed that the existence of alternative prey did not affect the control effect of *H. axyridis* on *M. persicae*.

Studies on indirect interaction mediated by shared natural enemies mainly focused on two pests on one plant (Liu et al., 2006; Bompard et al., 2013; Jaworski et al., 2015), leading to a hard discrimination between indirect interactions and resource competition (Bompard et al., 2013). Noonburg and Byers (2005) showed that the negative competitive impact of invasive species on native species is more likely to be apparent competition than resource competition. Meanwhile, Jones et al. (2009) stated that the effect of resource competition is higher than that of apparent competition in ecosystem. In our research, two preys apparently do not experience resource competition, but only indirect interaction mediated by shared predators. If natural enemies can quickly gather in habitat patches containing two preys with high density, then short-term apparent competition will also appear, and its mechanism is controlled by spatial effects (Holt and Lawton, 1994). However, we failed to test spatial effects in this study. In addition, the environmental factors (e.g., fertilizer application and climate warming) could also modulate natural enemy-mediated indirect interactions between pests, which could trigger multiple indirect bottom-up effects and increase both interspecific competition and overall biocontrol service (Han et al., 2019; Han et al., 2020; Han et al., 2022).

The establishment of biological control agents in the banker plant system is a probabilistic event that depends on the size of the founder population and the banker plant species (Sanchez et al., 2021). Compared with other studies in which mix-stage predators were introduced (Messelink et al., 2008; Bompard et al., 2013; Jaworski et al., 2015; Han et al., 2020), only one pair of *H. axyridis* adults was released. Allee effects had been suggested, which led to the decline of the suitability of predator population in the banker plant system (Sanchez et al., 2021). Therefore, the possible Allee effects may account for the differences in the effect of banker the plant system on *H. axyridis* abundance. Moreover, the timely replacement of banker plant may enhance the establishment of biological control agents in the banker plant system. Studies have proved that early planting and timely replanting of functional plants (e.g., repellent plants) in crops would ensure a more effective and suitable pest control (Hatt et al., 2019a; Wang et al., 2022).

Studies have shown that it is difficult to distinguish the negative effects of experiments from the operational problems related to complex experimental design, and it is difficult to detect the strongly apparent competition effect through experiments (Chaneton and Bonsall, 2000). Consequently, apparent competition and mutualism can be methodologically dissected in this food web when the banker plant system was applied to aphid control. This work adds to the previous literature on indirect food web interactions and the potentially indirect benefits of the banker plant system in pest management programs (Chailleux et al., 2014). On the basis of the co-effects of different initial density of alternative prey on target pest and the proliferation of predator, *H. axyridis*, we put forward two strategies for pest biological control. When the density of target prey is low, higher density of alternative prey in the banker plant system could enhance population buildup of beneficials without affecting the control effect on pest. When the density of target prey is high, lower density of alternative prey could directly support population of beneficials and facilitate indirectly the potential negative effect (i.e., apparent competition) on target pest in the ecosystem. Overall, we put forward a strategy for setting the initial density of alternative prey of the banker plant system to target the high and low density of aphids, which highlighted the importance of indirect interactions in designing a proper banker plant system.

## Data availability statement

The raw data supporting the conclusions of this article will be made available by the authors, without undue reservation.

## Author contributions

SW, SL, and JW conceived the research. JW, SL, and YY performed the experiments. JW, SL, and SW analyzed the data. JW, SL, YY, YL, ZJ, ND, PH, and SW wrote the manuscript. All authors contributed to the article and approved the submitted version.
