# Supplementary material for: Direct and indirect effects of banker plants on population establishment of Harmonia axyridis and aphid control on pepper crop
Source: Front Plant Sci. 2022 Dec 12;13:1083848. doi: 10.3389/fpls.2022.1083848 (PMC9792147; doi:10.3389/fpls.2022.1083848)
Supplement: Supplementary file 1 [file DataSheet_1.docx]

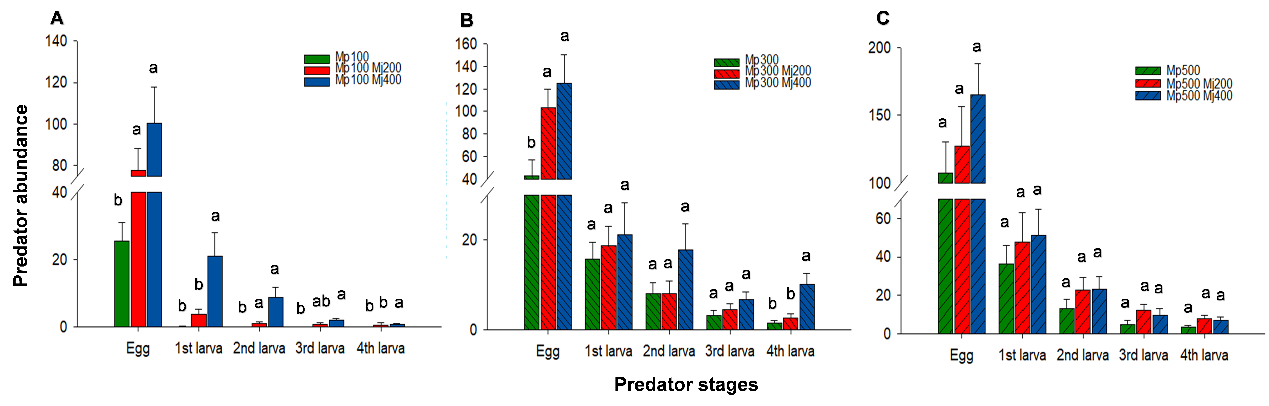


FIGURE S1 Cumulative output of *Harmonia axyridis* in different treatments with the initial density of *M. persicae* is 100 (A), 300 (B) and 500 (C). Different letters next to the curves indicate significant differences among treatments with ANOVA (*P*<0.05).


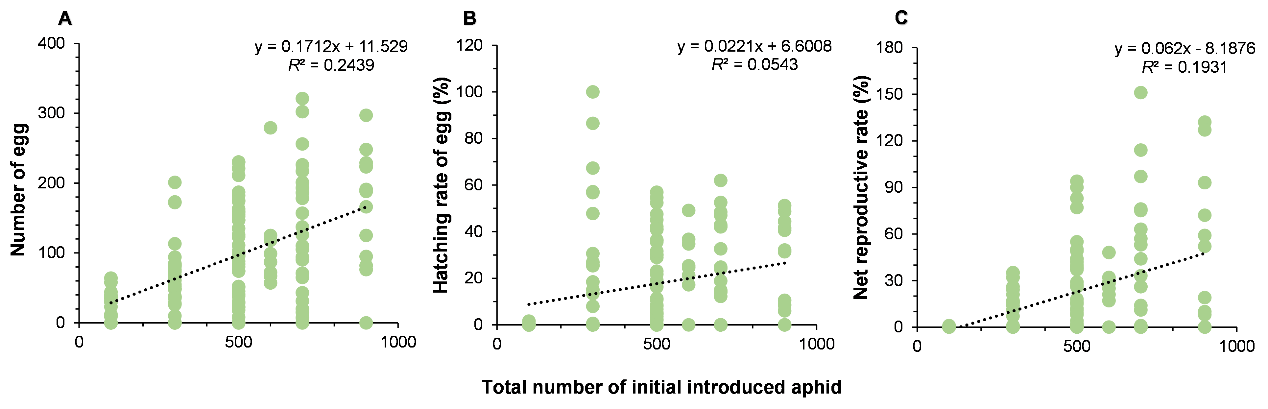


FIGURE S2 Correlation between the initial population of aphids and egg production (A), hatching rate of egg (B) and net reproductive rate (C) of *H**armonia axyridis* in different treatments.
